# Early childhood neurodevelopmental outcome after open prenatal spina bifida aperta repair

**DOI:** 10.1111/dmcn.14993

**Published:** 2021-07-23

**Authors:** Zehra S Hepp, Verena M Haas, Beatrice Latal, Martin Meuli, Ueli Möhrlen, Sonja M Schauer, Robert Steinfeld, Beth A Padden, David A Wille

**Affiliations:** ^1^ Division of Child Developmental Medicine University Children’s Hospital Zurich Zurich Switzerland; ^2^ Division of Pediatric Neurology University Children’s Hospital Zurich Zurich Switzerland; ^3^ Zurich Center for Spina Bifida University Children’s Hospital Zurich Zurich Switzerland; ^4^ Department of Pediatric Surgery University Children’s Hospital Zurich Zurich Switzerland; ^5^ The Zurich Center for Fetal Diagnosis and Therapy Zurich Switzerland; ^6^ Division of Pediatric Rehabilitation University Children’s Hospital Zurich Zurich Switzerland; ^7^ Department of Pediatric Neurology Kantonsspital Baden Baden Switzerland

## Abstract

**Aim:**

To investigate neurodevelopmental outcome of children with open prenatal spina bifida aperta (SBA) repair.

**Method:**

Prenatal SBA repair was performed in 130 fetuses at the Zurich Center between 2010 and 2019. Seventy‐seven children underwent 1 year assessment with the Griffiths Mental Developmental Scales (Griffiths) and 65 with the Bayley Scales of Infant and Toddler Development, Third Edition (Bayley‐III) at 2 years. Anatomical and functional level and ambulation status were assessed. Descriptive statistics and multiple linear regression analyses for risk factors were performed.

**Results:**

The Bayley‐III cognition composite score in children with prenatal SBA repair was within normal limits but lower compared to population norms (mean=95.15, SD=14.683 vs norm=100, SD=15, *p*=0.01). Fine motor development (mean=9.58, SD=2.744, *p*=0.227) was typical while gross motor development was lower than the norm (mean=3.02, SD=2.758 vs norm=10, SD=3, *p*<0.001). Griffiths developmental quotient subscales correlated significantly with corresponding Bayley‐III scores (all *p*<0.001, *r*=0.519–0.594). At 2 years, 50.8% could walk.

**Interpretation:**

Children with non‐trial open prenatal SBA repair show favourable cognitive outcome in the low‐average range at 1 and 2 years of age. While gross motor function remained delayed, fine motor function was age appropriate. The correlation between Griffiths and Bayley‐III allows a prediction about neurodevelopmental outcome at the age of 1 year.

**What this paper adds**

- Children with non‐trial open prenatal spina bifida repair show favourable cognitive outcome.
- Gross motor function remains impaired, while fine motor function is age appropriate.
- At 2 years of age, 50.8% of children were walking.
- Neurodevelopmental testing correlated between 1 (Griffiths Mental Developmental Scales) and 2 (Bayley Scales of Infant and Toddler Development, Third Edition) years.

## What this paper adds

- Children with non‐trial open prenatal spina bifida repair show favourable cognitive outcome.
- Gross motor function remains impaired, while fine motor function is age appropriate.
- At 2 years of age, 50.8% of children were walking.
- Neurodevelopmental testing correlated between 1 (Griffiths Mental Developmental Scales) and 2 (Bayley Scales of Infant and Toddler Development, Third Edition) years.

This original article is commented by Joyeux and Belfort on page 1243 of this issue.

**Abbreviations**
Bayley‐III: Bayley Scales of Infant and Toddler Development, Third Edition
CCS: Cognition composite score
Griffiths: Griffiths Mental Developmental Scales
LCS: Language composite score
LVB: Lumbar vertebral body
MCS: Motor composite score
MOMS: Management of Myelomeningocele Study
SBA: Spina bifida aperta

Spina bifida is a congenital abnormality of the central nervous system associated with long‐term morbidity.^1^, ^2^, ^3^ Spina bifida aperta (SBA) results from failed closure of the neural tube during early gestation,^4^ resulting in protrusion of the spinal cord and pia mater through a defect of the vertebral arches.^5^ Beside the early structural defect, exposure of neurological tissue to amniotic fluid inflicts progressive intrauterine damage to the spinal cord leading to lifelong neurodevelopmental sequelae.^6^

In 1995, Meuli et al.^7^ performed an iatrogenic spina bifida‐like lesion in fetal sheep, illustrating the damaging effect of amniotic fluid and traumatic factors on neural tissue. Further investigations showed that early intervention covering the defect led to decreased damage in the sheep model.^4^, ^5^ These findings paved the way to the ‘two‐hit hypothesis’, the first hit being failure of neurulation and the second chronic exposure of neural tissue to amniotic fluid.^4^

After promising results in animal studies, the first open prenatal repair of SBA was reported in 1998.^6^ The procedure was shown to prevent a remarkable degree of the neurological damage caused by intrauterine exposure to the amniotic fluid.^5^, ^8^, ^9^

As benefits and risks were still uncertain, the Management of Myelomeningocele Study (MOMS) was started in 2003 to examine the safety and efficacy of prenatal surgery for unborn fetuses with spina bifida. In 2010, the trial was stopped prematurely because of the significantly superior results of prenatal surgery, including improved motor function and increased likelihood for independent ambulation at the age of 30 months.^3^ This milestone trial suggested that, despite maternal and fetal risks, open prenatal repair might become a novel standard of care option for selected patients with SBA.^3^, ^9^, ^10^, ^11^

Previous studies have shown that children with SBA tend to have a neurocognitive function in the low‐average range^12^, ^13^ while motor function, including walking status without devices, is highly impaired.^3^, ^14^

Long‐term data of the MOMS cohort, published in 2020 by Houtrow et al.,^14^ showed no improvement in cognitive functioning in the prenatal repair group at primary school age compared to the postnatal group. In contrast, motor skills were improved in children with open prenatal SBA repair. However, there are persistent deficits in these children compared to typically developing children.^14^

In 2010, the Zurich Center for Fetal Diagnosis and Therapy was founded. To date, over 150 open prenatal SBA surgeries have been performed. The benefits of prenatal SBA surgery demonstrated by the MOMS trial were confirmed in a first benchmark analysis of the Zurich programme published in 2019 with comparable results regarding cognitive and motor function and ambulation status.^11^

This retrospective cohort study focuses on neurodevelopmental outcome in early childhood after open prenatal SBA repair in a centre which offers prenatal surgery as standard of care. While the MOMS trial published data from follow‐up assessment at 30 months, assessments of cognitive and motor function at the adjusted ages of 1 and 2 years are standard in many centres and our outcome is presented accordingly. We hypothesized that cognitive development would be in the low‐average range and motor skills would be below age norms at 2 years of age compared to population norms. Further we postulated that Griffiths Mental Developmental Scales (Griffiths) results at 1 year would correlate with the Bayley Scales of Infant and Toddler Development, Third Edition (Bayley‐III) results at 2 years of age.

## METHOD

### Study population

This is a retrospective cohort study including all children undergoing open prenatal SBA repair between December 2010 and December 2019 at the Zurich Center for Fetal Diagnosis and Therapy. The MOMS inclusion and exclusion criteria were applied with some modifications, as well as the criteria for hydrocephalus intervention.^3^ We continuously collected the same variables with additional modifications.

All patient families were asked to return for comprehensive multidisciplinary standardized assessments at the Zurich Center for Spina Bifida at the age of 3, 6, 9, 12, 18, and 24 months and annually thereafter until 18 years of age.

### Study design

Motor milestones in each child were examined by the same paediatric neurologists and rehabilitation specialists, while cognitive development was examined by a child development specialist. Since neurodevelopmental follow‐up is performed routinely as part of a clinical follow‐up programme, involved specialists were not blinded. Neurodevelopmental assessment was performed at the adjusted age of 1 year using the British norms of the Griffiths.^15^ At the adjusted age of 2 years, the Bayley‐III were administered.^16^ For better international comparison, US norms were applied.

Ambulation status at 2 years was assessed and divided into three subgroups: no walking, walking with orthotics or devices, and walking without orthotics.

The Griffiths assess five areas: locomotor, personal‐social, hearing and speech, eye and hand coordination, and performance. Results are declared in months. The Bayley‐III has five subscales, three as composite scores: cognition composite score (CCS), language composite score (LCS), and motor composite score (MCS), each with a mean of 100 and SD of 15 (<85 being mildly and <70 being significantly delayed). Furthermore, the test yields five subscales: cognition, receptive language, expressive language, fine motor function, and gross motor function, each with a mean of 10 and SD of 3.

In order to compare 1‐year Griffiths with 2‐year Bayley‐III assessments, a developmental quotient was calculated for each domain of the Griffiths (developmental age in relation to adjusted chronological age).

In our study, CCS of the Bayley‐III was compared to Griffiths performance and LCS to hearing and speech. Further, the gross motor subscale of the Bayley‐III was compared to Griffiths locomotor, and the Bayley‐III fine motor subscale to Griffiths eye and hand coordination.

Anatomical level was assessed by magnetic resonance imaging and defined as the first cranial open posterior vertebral arch. Functional level was determined by a paediatric neurologist depending on motor function of the lower extremities. It was defined as the lowest myotome with full muscle strength (strength of M5). Every reduced muscle activity (M1–M4) was considered motor partial innervation below the functional level according to the standard neurological examination of spinal cord injury.^17^ To distinguish clearly between anatomical and functional level, different wording was chosen: lumbar vertebral body (LVB) for anatomical and lumbar (L) for functional level. Our primary outcome was the cognitive outcome at 2 years (CCS). As secondary outcomes we defined the results of the Griffiths, ambulation status, MCS, LCS, and the correlation between the Griffiths and Bayley‐III as well as the correlation between level and ambulation status.

As potential influencing factors we defined sex, gestational age, ventriculoperitoneal shunt placement, endoscopic third ventriculostomy, and anatomical level.

Strengthening the Reporting of Observational Studies in Epidemiology (STROBE) guidelines for cohort studies were used.^18^

### Statistical analysis

Statistical analyses were performed with SPSS version 26.0 for Windows (IBM Corp., Armonk, NY, USA) and R version 3.6.2 (R Foundation for Statistical Computing, Vienna, Austria).

Descriptive statistics were used to record demographic and clinical characteristics. Results are expressed as mean and SD. A multiple linear regression analysis was used to assess potential risk factors while controlling for potential confounders. Comparison between groups for continuous variables was performed using *t‐*tests and Mann–Whitney *U* tests, depending on the sample distribution. Because the majority of variables showed non‐normal distribution according to the Kolmogorov–Smirnov test, we used nonparametric tests for all variables testing equality of means; only the results of the Griffiths were normally distributed and therefore *t*‐test was used. We tested for homoscedasticity for the Griffiths results. In the eye and hand coordination subtest the homoscedasticity was not given; therefore we reported the *p*‐value for non‐equal variance in this subtest. Comparisons between groups and a norm value were performed using Welch’s modified two‐sample *t*‐test. Comparisons for categorical variables were assessed by χ^2^ test. Correlation between Griffiths and Bayley‐III was performed with Spearman’s rank correlation coefficient. All statistical tests were two‐sided and a *p*‐value of less than 0.05 was considered significant.

### Ethics and informed consent

The study was approved by the ethical committee of the Canton of Zurich (KEK‐ZH No. 2015‐0172). All parents or legal guardians gave written informed consent before participating in the study and publication of the results.

## RESULTS

### Study population and baseline characteristics

From December 2010 to December 2019, 130 children underwent prenatal surgery, 124 were born by 31st December 2019 (Fig. S1, online supporting information). Two children were excluded from the data set because of intrauterine and perinatal death. Ten children were lost to follow‐up and excluded because of relocations of the families, poor condition of the child, living abroad, or other reasons.

One‐ and 2‐year outcomes are based on 77 and 65 children who had reached the adjusted age of 1 year by 31st December 2019 and the age of 2 years by 11th September 2020 respectively, and had complete follow‐up data. Twenty‐one children had incomplete follow‐up data because of crying, tiredness, or time management problems on the day of examination.

Comparison of baseline characteristics at 1 and 2 years of age between those with and without follow‐up (too young or lost to follow‐up) showed no difference (Tables S1 and S2, online supporting information).

### Neurodevelopmental outcome at 1 and 2 years of age

At 1 year, mean Griffiths developmental quotient scores and SD were assessed for all subscales (Table 1). Children with prenatal SBA repair performed worse than the norm in all subscales, especially in the locomotor subscale.

**Table 1:** Neurodevelopmental outcome at the age of 1 and 2 years

Assessment	Mean	SD	*p*	Norm
Griffiths scales at 1 year				
Locomotor developmental quotient	67	14	<0.001	100^a^
Personal‐social developmental quotient	86	12	<0.001	100^a^
Hearing and speech developmental quotient	85	14	<0.001	100^a^
Eye and hand coordination developmental quotient	84	12	<0.001	100^a^
Performance developmental quotient	86	12	<0.001	100^a^
Bayley‐III at 2 years				
Cognition composite score	95.15	14.683	0.01	100^a^
Cognition scaled score	9.05	2.930	0.011	10^b^
Language composite score	89.83	13.266	<0.001	100^a^
Language receptive scaled score	8.65	2.335	<0.001	10^b^
Language expressive scaled score	7.86	2.651	<0.001	10^b^
Motor composite score	77.78	13.509	<0.001	100^a^
Fine motor scaled score	9.58	2.744	0.227	10^b^
Gross motor scaled score	3.02	2.758	<0.001	10^b^

Neurodevelopmental performance on the Griffiths Mental Developmental Scales (Griffiths) at the age of 1 year (*n*=77) and on the Bayley Scales of Infant and Toddler Development, Third Edition (Bayley‐III) at the age of 2 years (*n*=65) including receptive and expressive language and fine and gross motor function, showing poorer outcomes over all areas in children with prenatal spina bifida aperta repair compared to population norms except for fine motor function in the Bayley‐III. ^a^Standard deviation (SD)=15.^b^SD=3

At 2 years of age, mean CCS and LCS were in the low‐average range and significantly lower than the norm. MCS was significantly lower compared to the population norm. When looking at the subscales, gross motor function was significantly worse than in the typically developing population, while no significant difference for fine motor function was found.

In population norms, 15.87% are expected to score below 85 in the Bayley‐III. In our cohort, the percentage proportion was 16.9% (*n*=11) for the CCS (*p*=0.816), 30.8% (*n*=20) for the LCS (*p*=0.001), and 69.2% (*n*=45) for the MCS (*p*<0.001). A score below 70 is expected in 2.28% of population norms. In children with prenatal SBA repair, the percentage proportion was 3.1% (*n*=2) for the CCS (*p*=0.667), 4.6% (*n*=3) for the LCS (*p*=0.207), and 20% (*n*=13) for the MCS (*p*<0.001).

We investigated risk factors for neurodevelopmental outcome at the age of 2 years, such as gestational age, sex, ventriculoperitoneal shunt placement, endoscopic third ventriculostomy, and anatomical level. Birthweight was not included in the model because of strong intercorrelation (*r*=0.766) with gestational age. When the above variables were included in a multiple linear regression model, only patients with increasing hydrocephaly and indication for ventriculoperitoneal shunt placement performed poorer in CCS (*p*=0.003, regression coefficient *B*=–11.609). When functional level was added to the model, results were similar (Table S3, online supporting information).

When comparing neurodevelopmental scores between the age of 1 (Griffiths) and 2 years (Bayley‐III), a statistically significant correlation was found as follows: Griffiths hearing and speech developmental quotient and LCS (*p*<0.001, *r*=0.549), Griffiths performance developmental quotient and CCS (*p*<0.001, *r*=0.519), Griffiths eye and hand coordination developmental quotient and Bayley‐III fine motor (*p*<0.001, *r*=0.571), and Griffiths locomotor developmental quotient and Bayley‐III gross motor (*p*<0.001, *r*=0.594).

### Neuromotor development at 2 years of age

Neuromotor assessment at 2 years included anatomical level, functional level, motor partial innervation, and ambulation status (Table 2). The percentage portion of anatomical level LVB4 and LVB5 was greater than 50%. Assessment of the functional level showed that 33.9% had a level of L4, 50.8% a level of L5, and 4.6% a level of sacral (S) 1. A motor partial innervation was present in 96.9%; in 87.3% it reached level S1. Investigation of ambulation status showed that at the age of 2 years, 50.8% (33/65) walked with or without orthotics or devices. The percentage of above‐mentioned walkers with an anatomical level of LVB4 and LVB5 was 44.4% and 60% respectively, while with a functional level of L4 and L5, 31.8% and 66.7% were walkers respectively. A correlation between anatomical level and ambulation status showed that children with a lower level tend to have a higher possibility of walking (*p*=0.004).

**Table 2:** Level and ambulation status at the age of 2 years

Level specification	Children, *n* (%)	Ambulation,^a^ *n* (%)
Anatomical level		
TVB11	1 (1.5)	0 (0)
TVB12	1 (1.5)	1 (100)
LVB1	3 (4.62)	0 (0)
LVB2	4 (6.15)	1 (25)
LVB3	9 (13.85)	3 (33.33)
LVB4	18 (27.7)	8 (44.44)
LVB5	20 (30.77)	12 (60)
SVB1	9 (13.85)	8 (88.89)
Total	65 (100)	33 (50.77)
Functional level		
L2	1 (1.5)	1 (100)
L3	3 (4.62)	0 (0)
L4	22 (33.85)	7 (31.82)
L5	33 (50.77)	22 (66.67)
S1	3 (4.62)	1 (33.33)
S2	1 (1.5)	1 (100)
Unknown	2 (3.08)	1 (50)
Total	65 (100)	33 (50.77)

Overview of anatomical and functional level and ambulation status at the age of 2 years (*n*=65). ^a^Ambulation: includes walking with or without orthotics or devices. TVB, thoracic vertebral body; LVB, lumbar vertebral body; SVB, sacral vertebral body; L, lumbar; S, sacral.

## DISCUSSION

The Zurich cohort of children with prenatal SBA repair is one of the largest in the world. This study provides outcome data from a centre offering fetal surgery as a standard procedure.

Our hypothesis that children with prenatal SBA repair have a favourable cognitive outcome in the low‐average range both at 1 and 2 years of age was confirmed. Additionally, motor function was significantly below age‐appropriate norms at 2 years as expected. A correlation between Griffiths and Bayley‐III was confirmed.

Generally, children with SBA are known to have low‐average neurocognitive function.^12^, ^13^ The MOMS trial demonstrated that prenatal SBA repair did not significantly alter cognitive functioning compared to the postnatal cohort. In the trial, similar low‐average cognitive scores were found for the prenatal and postnatal group at 30 months and between 5 and 10 years of age.^3^, ^14^ These results were confirmed by our study: mean cognitive and language performance at 2 years of age were within the low‐average range.

Various factors may impact cognitive outcome in children with prenatal SBA repair. We found that cognition at the age of 2 years was lower in children after shunting, but literature on the effect of ventriculoperitoneal shunt placement on cognitive outcome is inconsistent. A meta‐analysis performed by Johnson et al.^19^ showed that shunt placement negatively impacts cognitive outcome. In contrast, another recent meta‐analysis showed no difference in cognition between shunted and not shunted children with prenatal SBA repair.^20^ It is conceivable that the indication for ventriculoperitoneal shunt placement differs between studies. Further investigations are needed to evaluate the effect of hydrocephalus and shunt placement on cognitive outcome. Another study from our centre will focus on the role of ventriculomegaly and cerebrospinal fluid diversion in correlation to development.

Another risk factor for cognitive impairment is anatomical level. In our cohort, the majority of children had a lumbar level. Our findings suggest that there is no correlation between anatomical level and CCS. Previous findings are inconsistent. Johnson et al.^19^ had results similar to ours. Their focus was on 51 children at 2 years with prenatal SBA repair, the majority with lumbar lesions. Some studies, however, suggest that only higher levels have a significant negative impact on cognitive outcome.^21^, ^22^ Fletcher et al.^21^ showed that higher anatomical levels were not only associated with lower cognitive outcome, but also with a higher prevalence of brain abnormalities which could contribute to poorer cognitive outcome.

In contrast to the favourable cognitive outcomes, motor function was statistically significantly impaired. A mean of 77.78 is considered to have a high clinical impact with relevant motor disabilities. Despite improvements in physical functioning in children with prenatal compared to postnatal SBA repair, children with prenatal SBA repair still demonstrate deficits in motor development.^3^ When dividing motor function at the age of 2 years into fine and gross motor function, only gross motor function was impaired. An explanation for more favourable fine motor function compared to gross motor function is that assessment of motor function at the age of 2 years focuses on gross motor milestones, while fine motor requirements are limited at this age and standardized tests are more thorough at an older age.^23^, ^24^ Detailed assessment of motor function improves with age.

Evaluation of ambulation status at 2 years of age showed a delay compared to typically developing children. While in the general population over 90% are walking at the age of 2 years, in our cohort 50.8% could walk (with or without orthotics or devices).^25^ Preliminary analysis show that these rates increase up to nearly 90% by 3 years (unpublished data). Previous studies from the MOMS cohort report a significantly higher rate of walkers (with or without orthotics or devices) in children with prenatal rather than postnatal SBA repair at 30 months (71% vs 57%) as well as between 5 and 10 years of age (93% vs 80%).^3^, ^14^ Danzer et al.^26^, ^27^ showed enduring results regarding independent ambulation in older children.

An instrumental question focused on the connection between ambulation and anatomical level, which showed that lower anatomical levels are related to a higher rate of ambulation. At age 2 years, 44.4% with an anatomical level of LVB4 and 60% with LVB5 were ambulating. These findings are similar when looking at functional levels. At age 2 years, 31.8% with L4 and 66.7% with L5 were walking with or without orthotics or devices. This difference can be partially explained with neurophysiological knowledge about gait and the high rate of partial innervation to S1 (87.3%). The hamstring muscles are an important component of sufficient walking. For full strength, an intact spinal nerve innervating the myotome L5 is necessary. The plantar flexors and hip extenders, both S1 muscles, are essential for independent ambulation.^28^

We also report that developmental scores assessed with the Griffiths subscales at 1 year showed a strong correlation with the Bayley‐III subscales at 2 years. These findings confirm our hypothesis and suggest that the Griffiths are appropriate screening tools for neurodevelopmental outcome. This correlation can assist clinicians in counselling parents about expected developmental outcome at the age of 2 years and initiating special needs therapy.

The main limitation is that outcome is not compared with postnatal SBA repair. Moreover, results can only be applied to other centres with similar procedures and patient characteristics. Our study includes a diverse population from Europe and Russia; therefore the results may differ in other ethnic groups.

In summary, the Zurich Center harbours a large non‐trial cohort with an extensive follow‐up programme. Cognitive outcome in children with open prenatal SBA repair at 2 years is within the low‐average range. Motor function was impaired compared to typically developing children, especially regarding gross motor function leading to delayed ambulation at 2 years. Further investigations must evaluate these findings over time. Detailed long‐term follow‐up is essential in order to provide appropriate medical care and counselling prenatally and during development.

## Supporting information


**Figure S1:** Overview of all participants included in this study and the follow‐up

**Table S1:** Baseline characteristics of the study population at the age of 1 year

**Table S2:** Baseline characteristics of the study population at the age of 2 years

**Table S3:** Multiple linear regression for risk factor analysis for neurodevelopmental outcome at the age of 2 years.

## Data Availability

Data available on request from the authors.
