# Supplementary figures and images for: Early childhood neurodevelopmental outcome after open prenatal spina bifida aperta repair
Source: Dev Med Child Neurol. 2021 Jul 23;63(11):1302–7. doi: 10.1111/dmcn.14993 (PMC8596420; doi:10.1111/dmcn.14993)

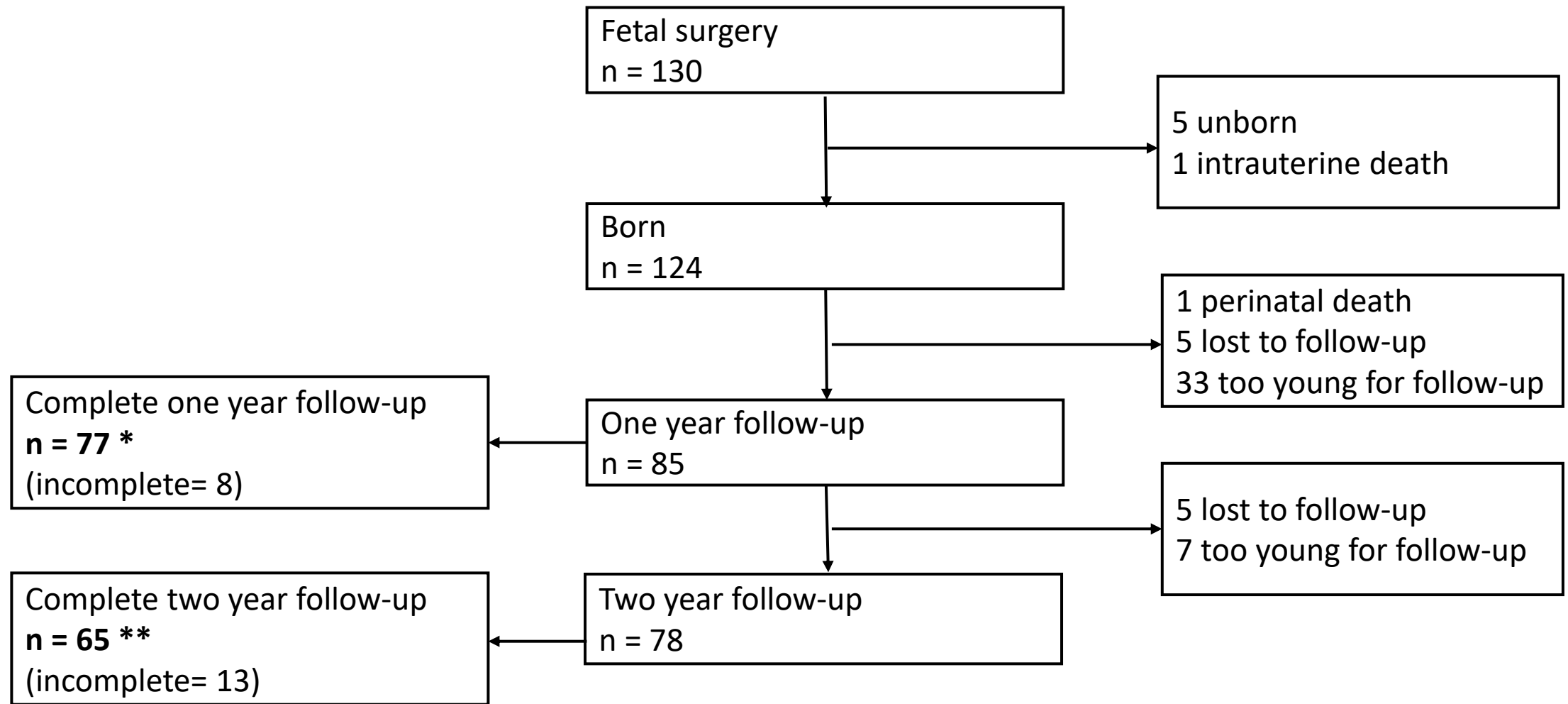

\* December 31, 2019. \*\* September 11, 2020.

Supplement: Supplementary file 1 — Figure S1: Overview of all participants included in this study and the follow‐up [file DMCN-63-1302-s003.pdf]
