# Supplementary material for: Early childhood neurodevelopmental outcome after open prenatal spina bifida aperta repair
Source: Dev Med Child Neurol. 2021 Jul 23;63(11):1302–7. doi: 10.1111/dmcn.14993 (PMC8596420; doi:10.1111/dmcn.14993)
Supplement: Supplementary file 2 — Table S1: Baseline characteristics of the study population at the age of 1 year [file DMCN-63-1302-s004.docx]

| **Supplemental Table I: Baseline characteristics of the study population at the age of one year *** | | | | | | | | | | |
| --- | --- | --- | --- | --- | --- | --- | --- | --- | --- | --- |
|  |  |  |  |  |  |  |  |  |  |  |
| **Parameter** | **All (*n*=115*)*** | | | **Seen (*n*=77)** | | | **Not seen (*n*=38)** | | | ***P* _seen vs. not seen_** |
|  |  |  |  |  |  |  |  |  |  |  |
|  |  |  |  |  |  |  |  |  |  |  |
|  | **Mean** | **SD** | **Range** | **Mean** | **SD** | **Range** | **Mean** | **SD** | **Range** |  |
| Gestational age at birth  (w + d) | 35 + 2 | 2 + 2 | 25+2 - 38+2 | 35 + 3 | 2 + 1 | 25+2 - 37+6 | 35 +3 | 2+2 | 28+4 - 38+2 | 0.842 |
| Birth weight (g) | 2564.17 | 510.292 | 850-3670 | 2558.39 | 473.86 | 850 - 3390 | 2575.87 | 583.838 | 1150 - 3670 | 0.338 |
| Maternal age at screening (y + m) | 31 + 3 | 5 + 0 | 22-45 | 31 + 1 | 4 + 11 | 22 - 41 | 32+0 | 5+2 | 23 - 45 | 0.404 |
|  |  |  |  |  |  |  |  |  |  |  |
|  |  |  |  |  |  |  |  |  |  |  |
|  | ***n* (%)** |  |  | ***n* (%)** | |  | ***n*** | |  |  |
| Female sex | 61 (52.1) |  |  | 44 (53.7) | |  | 19 | |  | 0.646 |
|  |  |  |  |  |  |  |  |  |  |  |
| **Shunt status** |  |  |  |  |  |  |  |  |  |  |
| VP Shunt | 31 (26.5) |  |  | 22 (26.8) | |  | 10 | |  | 0.913 |
| ETV | 7 (6.0) |  |  | 6 (7.3) | |  | 2 | |  | 0.795 |
| ETV + secondary shunt | 5 (4.3) |  |  | 5 (6.1) | |  | 0 | |  | 0.108 |
|  |  |  |  |  |  |  |  |  |  |  |
| **Anatomical level** |  |  |  |  |  |  |  |  |  |  |
| TVB 9 | 1 (0.9) |  |  | 0 (0) | |  | 1 | |  | 0.388 |
| TVB 10 | 1 (0.9) |  |  | 1 (1.2) | |  | 0 | |  |  |
| TVB 11 | 1 (0.9) |  |  | 1 (1.2) | |  | 0 | |  |  |
| TVB 12 | 1 (0.9) |  |  | 1 (1.2) | |  | 0 | |  |  |
| LVB 1 | 2 (1.7) |  |  | 2 (2.4) | |  | 0 | |  |  |
| LVB 2 | 6 (5.1) |  |  | 5 (6.1) | |  | 1 | |  |  |
| LVB 3 | 16 (13.7) |  |  | 11 (13.4) | |  | 5 | |  |  |
| LVB 4 | 27 (23.1) |  |  | 20 (24.4) | |  | 8 | |  |  |
| LVB 5 | 41 (35.0) |  |  | 30 (36.6) | |  | 14 | |  |  |
| SVB 1 | 15 (12.8) |  |  | 11 (13.4) | |  | 5 | |  |  |
| SVB 2 | 1 (0.9) |  |  | 0 (0) | |  | 1 | |  |  |
| Baseline characteristics of the children with complete one-year follow-up data compared to all eligible patients without completed follow-up. W=weeks, d=days, g=gram, y=years, m=months, VP=Ventriculoperitoneal, ETV=endoscopic third ventriculostomy, TVB=thoracic vertebral body, LVB=lumbar vertebral body, SVB=sacral vertebral body, SD=standard deviation. * Percentages may not be 100 because of rounding. | | | | | | | | | | |
