# Supplementary material for: Early childhood neurodevelopmental outcome after open prenatal spina bifida aperta repair
Source: Dev Med Child Neurol. 2021 Jul 23;63(11):1302–7. doi: 10.1111/dmcn.14993 (PMC8596420; doi:10.1111/dmcn.14993)
Supplement: Supplementary file 3 — Table S2: Baseline characteristics of the study population at the age of 2 years [file DMCN-63-1302-s001.docx]

| **Supplemental Table II: Baseline characteristics of the study population at the age of two years*** | | | | | | |  |  |  |  |
| --- | --- | --- | --- | --- | --- | --- | --- | --- | --- | --- |
|  |  |  |  |  |  |  |  |  |  |  |
| **Parameter** | **all (*n*=110)** | | | **Seen (*n*=65)** | | | **Not seen (*n*=45)** | | | ***P* _seen vs. not seen_** |
|  |  |  |  |  |  |  |  |  |  |  |
|  |  |  |  |  |  |  |  |  |  |  |
|  | **Mean** | **SD** | **Range** | **Mean** | **SD** | **Range** | **Mean** | **SD** | **Range** |  |
| Gestational age at birth (w + d) | 35 + 3 | 2 + 1 | 25+2 - 38+2 | 35 + 4 | 2 + 1 | 25.29-37.86 | 35 + 2 | 2 + 2 | 28+4-38+2 | 0.469 |
| Birth weight (g) | 2572.63 | 511.319 | 850-3670 | 2610.46 | 478.3 | 850-3390 | 2517.98 | 556.551 | 1150-3670 | 0.607 |
| Maternal age at screening (y + m) | 31 + 3 | 5 + 0 | 22-45 | 31 + 1 | 4 + 4 | 22-40 | 32 + 0 | 5 + 5 | 23-45 | 0.489 |
|  |  |  |  |  |  |  |  |  |  |  |
| **Griffiths °** |  |  |  |  |  |  |  |  |  |  |
| Locomotor DQ | 68.43 | 14.721 |  | 68.85 | 14.427 |  | 65.24 | 17.554 |  | 0.514 |
| Personal-Social DQ | 85.28 | 13.25 |  | 84.84 | 13.506 |  | 89.16 | 10.753 |  | 0.413 |
| Hearing and Speech DQ | 85.1 | 14.103 |  | 85.11 | 14.652 |  | 85.06 | 8.473 |  | 0.993 |
| Eye and Hand Coordination DQ | 83.73 | 12.34 |  | 83.81 | 12.825 |  | 82.99 | 7.278 |  | 0.868 |
| Performance DQ | 85.16 | 12.169 |  | 84.95 | 12.275 |  | 86.97 | 11.935 |  | 0.677 |
|  |  |  |  |  |  |  |  |  |  |  |
|  | ***n* (%)** |  |  | ***n* (%)** |  |  | ***n*** |  |  |  |
| Female sex | 61 (55.5) |  |  | 37 (56.9) |  |  | 24 |  |  | 0.711 |
|  |  |  |  |  |  |  |  |  |  |  |
| **Shunt status** |  |  |  |  |  |  |  |  |  |  |
| VP Shunt | 33 (30) |  |  | 21 (32.3) |  |  | 12 |  |  | 0.527 |
| ETV | 5 (4.5) |  |  | 3 (4.6) |  |  | 2 |  |  | 0.966 |
| ETV + secondary shunt | 6 (5.5) |  |  | 4 (6.2) |  |  | 2 |  |  | 0.699 |
|  |  |  |  |  |  |  |  |  |  |  |
| **Anatomical level** | |  |  |  |  |  |  |  |  |  |
| TVB 9 | 1 (0.9) |  |  | 0 (0) |  |  | 1 |  |  | 0.664 |
| TVB 10 | 2 (1.8) |  |  | 0 (0) |  |  | 2 |  |  |  |
| TVB 11 | 1 (0.9) |  |  | 1 (1.5) |  |  | 0 |  |  |  |
| TVB 12 | 2 (1.8) |  |  | 2 (3.1) |  |  | 0 |  |  |  |
| LVB 1 | 3 (2.7) |  |  | 2 (3.1) |  |  | 1 |  |  |  |
| LVB 2 | 7 (6.4) |  |  | 4 (6.2) |  |  | 3 |  |  |  |
| LVB 3 | 15 (13.6) |  |  | 9 (13.8) |  |  | 6 |  |  |  |
| LVB 4 | 26 (23.6) |  |  | 18 (27.7) |  |  | 8 |  |  |  |
| LVB 5 | 37 (33.6) |  |  | 20 (30.8) |  |  | 17 |  |  |  |
| SVB 1 | 15 (13.6) |  |  | 9 (13.8) |  |  | 6 |  |  |  |
| SVB 2 | 1 (0.9) |  |  | 0 (0) |  |  | 1 |  |  |  |
| Baseline characteristics of the children with complete two-year follow-up data compared to all eligible patients without completed follow-up. W=weeks, d=days, g=gram, y=years, m=months, DQ=developmental quotient, VP=ventriculoperitoneal, ETV=endoscopic third ventriculostomy, TVB=thoracic vertebral body, LVB=lumbar vertebral body, SVB=sacral vertebral body, SD=standard deviation. * Percentages may not be 100 because of rounding. ° *n*=67-69 for the group "all", *n*=60-62 for the group "seen" and *n*=7-8 for the group "not seen". | | | | | | | | | | |
