# Supplementary material for: Early childhood neurodevelopmental outcome after open prenatal spina bifida aperta repair
Source: Dev Med Child Neurol. 2021 Jul 23;63(11):1302–7. doi: 10.1111/dmcn.14993 (PMC8596420; doi:10.1111/dmcn.14993)
Supplement: Supplementary file 4 — Table S3: Multiple linear regression for risk factor analysis for neurodevelopmental outcome at the age of 2 years. [file DMCN-63-1302-s002.docx]

| **Supplemental Table III: Multiple linear regression for risk factor analysis for neurodevelopmental outcome at the age of two years** | | | | | | | | | | |
| --- | --- | --- | --- | --- | --- | --- | --- | --- | --- | --- |
|  |  |  |  |  |  |  |  |  |  |  |
| **Dependent variable** | | | **Independent variable** |  |  | ***B*(95% CI)** |  | **SD** | ***β*** | ***p*-value** |
| Cognition Composite Score |  |  | Constant |  |  | 99.321 (20.513-178.129) |  | 39.385 |  | 0.014 |
|  | | | Male |  |  | -4.094 (-11.541-3.353) |  | 3.722 | -0.139 | 0.276 |
|  |  |  | Gestational age |  |  | -0.184 (-1.869-1.5) |  | 0.842 | -0.027 | 0.827 |
|  | | | Anatomical lesion level |  |  | 0.364 (-1.945-2.673) |  | 1.154 | 0.039 | 0.754 |
|  | | | Ventriculoperitoneal shunting |  |  | -11.609 (-19.005- -4.213) |  | 3.696 | -0.385 | 0.003 |
|  | | | Endoscopic third ventriculostomy |  |  | 0.577 (-11.064-12.218) |  | 5.817 | 0.012 | 0.921 |
|  |  |  |  |  |  |  |  |  |  |  |
| Language Composite Score | | | Constant |  |  | 123.332 (48.968-197.695) |  | 37.163 |  | 0.002 |
|  |  |  | Male |  |  | -3.062 (-10.089-3.965) |  | 3.512 | -0.115 | 0.387 |
|  |  |  | Gestational age |  |  | -0.882 (-2.472-0.707) |  | 0.794 | -0.143 | 0.271 |
|  | | | Anatomical lesion level |  |  | 0.026 (-2.205-2.152) |  | 1.089 | -0.003 | 0.981 |
|  |  |  | Ventriculoperitoneal shunting |  |  | -2.433 (-9.412-4.546) |  | 3.488 | -0.089 | 0.488 |
|  | | | Endoscopic third ventriculostomy |  |  | 6.782 (-4.202-17.766) |  | 5.489 | 0.16 | 0.222 |
|  | | |  |  |  |  |  |  |  |  |
| Motor Composite Score | | | Constant |  |  | 37.160 (-36.458-110.779) |  | 36.791 |  | 0.317 |
|  | | | Male |  |  | -0.403 (-7.359-6.554) |  | 3.477 | 0.015 | 0.908 |
|  | | | Gestational age |  |  | 0.188 (-1.386-1.761) |  | 0.786 | 0.03 | 0.812 |
|  | | | Anatomical lesion level |  |  | 1.62 (-0.536-3.777) |  | 1.078 | 0.187 | 0.138 |
|  | | | Ventriculoperitoneal shunting |  |  | -8.081 (-14.99- -1.171) |  | 3.453 | -0.291 | 0.023 |
|  | | | Endoscopic third ventriculostomy |  |  | -2.015 (-12.889-8.859) |  | 5.434 | -0.047 | 0.712 |
| Multiple linear regression analysis for risk factor analysis for neurodevelopmental outcome at the age of two years. Cognition composite score: *R2* = 0.162, *p*=0.059. Language Composite score: *R2* = 0.085, *p*=0.369. Motor Composite score: *R2* = 0.136, *p*=0.117. *B* = Regression weight, 95% CI = 95% confidence intervals, SD= Standard deviation, *β* = Standardized regression weight | | | | | | | | | | |
